# Ambient but not local lactate underlies neuronal tolerance to prolonged glucose deprivation

**DOI:** 10.1371/journal.pone.0195520

**Published:** 2018-04-04

**Authors:** Courtney Sobieski, Natasha Warikoo, Hong-Jin Shu, Steven Mennerick

**Affiliations:** 1 Department of Psychiatry, Washington University School of Medicine, St. Louis, MO, United States of America; 2 Graduate Program in Neuroscience, Washington University School of Medicine, St. Louis, MO, United States of America; 3 Taylor Family Institute for Innovative Psychiatric Research, Washington University School of Medicine, St. Louis, MO, United States of America; 4 Department of Neuroscience, Washington University School of Medicine, St. Louis, MO, United States of America; Albany Medical College, UNITED STATES

## Abstract

Neurons require a nearly constant supply of ATP. Glucose is the predominant source of brain ATP, but the direct effects of prolonged glucose deprivation on neuronal viability and function remain unclear. In sparse rat hippocampal microcultures, neurons were surprisingly resilient to 16 h glucose removal in the absence of secondary excitotoxicity. Neuronal survival and synaptic transmission were unaffected by prolonged removal of exogenous glucose. Inhibition of lactate transport decreased microculture neuronal survival during concurrent glucose deprivation, suggesting that endogenously released lactate is important for tolerance to glucose deprivation. Tandem depolarization and glucose deprivation also reduced neuronal survival, and trace glucose concentrations afforded neuroprotection. Mass cultures, in contrast to microcultures, were insensitive to depolarizing glucose deprivation, a difference attributable to increased extracellular lactate levels. Removal of local astrocyte support did not reduce survival in response to glucose deprivation or alter evoked excitatory transmission, suggesting that on-demand, local lactate shuttling is not necessary for neuronal tolerance to prolonged glucose removal. Taken together, these data suggest that endogenously produced lactate available globally in the extracellular milieu sustains neurons in the absence of glucose. A better understanding of resilience mechanisms in reduced preparations could lead to therapeutic strategies aimed to bolster these mechanisms in vulnerable neuronal populations.

## Introduction

The human brain represents only 2% of total body mass, yet it accounts for a disproportionately large amount of total energy consumption. The energy requirements of the mammalian brain are largely met by the metabolism of glucose. To produce ATP necessary for central nervous system function, glucose is broken down via glycolysis and the TCA cycle/oxidative phosphorylation (OXPHOS). Neurons are tasked with the upkeep of many energetically expensive functions such as maintaining ion gradients, generating and propagating action potentials, and fueling synaptic transmission, all of which require a significant amount of ATP[1]. Synaptic transmission is considered the most metabolically expensive neuronal function[2] and is especially sensitive to disruptions in glucose availability and subsequent ATP production. Neurons lose the ability to communicate within minutes of inhibiting ATP production[3–9]. Unlike neighboring astrocytes, neurons canonically do not possess glycogen[10] (though see[11,12]) and have limited phosphocreatine reserves to supply ATP. Thus, they rely heavily on the availability of extracellular metabolic substrates[13].

The cumulative effects of glucose deprivation have been previously studied, predominantly as a model for pathological conditions such as tight insulin control in diabetes or in the context of cerebral ischemia[14–16]. Brain hypoglycemia is associated with overstimulation of glutamate receptors and excitotoxic death of neurons[17–21]. This excitotoxicity is secondary to small decreases in ATP and clouds the core consequences of glucose deprivation on neuronal survival and signaling. Thus, although other studies have investigated the effects of acute glucose removal and alternative substrates on neuronal physiology[4–8,22–24], the direct impact of glucose deprivation on aspects of synaptic communication remain unclear. To isolate and manipulate neuron-glia interactions and explore susceptibility, a reductionist approach is warranted. In the present study, we use rat co-cultures of hippocampal neurons and astrocytes to investigate the effect of prolonged glucose deprivation on neuronal survival and synaptic function. To focus on core, local cellular interactions, we employed microcultures, local units of a few astrocytes and neurons, to probe fuel sources for neurons. Our results suggest that neuronal survival and synaptic function are both surprisingly resilient to prolonged loss of glucose. During glucose deprivation, OXPHOS is apparently adequately maintained in the absence of exogenous glucose to support survival and signaling. Although this resiliency is sustained predominately by ambient extracellular lactate derived from astrocytes, local on-demand lactate shuttling does not meaningfully contribute to the ATP generation that sustains survival and synaptic function.

## Materials and methods

### Hippocampal cell culture

Neuron-astrocyte co-cultures were created and maintained as previously described[25,26] Briefly, postnatal day 1–4 Sprague-Dawley rat hippocampal (neuron) and cortical (astrocyte) tissue were harvested using protocols approved by the Washington University Animal Studies Committee and in accordance with relevant guidelines and regulations. The tissue was digested by 1 mg/ml papain, and mechanically dispersed. For microculture preparations, astrocytes were first plated on top of collagen microdots in Eagle’s medium (Life Technologies) supplemented with 5% heat-inactivated horse serum, 5% fetal bovine serum, 17 mM D-glucose, 400 μM glutamine, 500 U/ml penicillin, and 50 μg/ml streptomycin. They were maintained at 37°C in a humidified incubator (5% CO_2_/95% air) and treated with 10 μM cytosine arabinoside to halt proliferation before neuronal plating. Neurons were plated at a low density (~100 cells/mm^-2^). Microcultures containing (+astrocyte) or lacking (-astrocyte) an astrocyte layer on the collagen microdot were prepared as previously described[27]. Briefly, 25-mm glass coverslips were stamped with a polydimethylsiloxane microstamp coated with 0.5 mg/ml collagen to create 150–200 μm diameter microdots. Coverslips were then backfilled with the non-permissive substrate poly-l-lysine grafted polyethylene glycol (PLL(20 kDa)-g[3.5]-PEG(2 kDa); Surface Solutions, Dübendorf, Switzerland) at 10 μg/ml in PBS for 1 hour and then washed with 1x PBS. A sample of +astrocyte microcultures, evaluated by Hoechst staining of nuclei, was found to have 12.3 ± 1.7 astrocytes per microculture, suggesting ample opportunity for local interaction with resident neurons. Mass cultures were prepared by seeding astrocytes and neurons (~650 cells/mm^2^) onto a coverslip coated with poly-D-lysine and laminin. Astrocyte-only mass cultures were produced by dissociating mass cultures of glia and neurons via trypsinization at DIV 5–6. These cultures were then allowed to recover for 6–7 days prior to removal of glucose. Unless otherwise stated, experiments were performed 9–14 days *in vitro*.

### Neuronal survival

To test the effects of prolonged glucose deprivation on neuronal survival, culture medium of microcultures and high density mass cultures were either removed and replaced with 1 mL of incubation saline (1x wash) or washed twice (15 s each) with fresh aliquots of 1 mL of incubation saline before final incubation in 1 mL of incubation saline (3x wash). We estimated that 99% of conditioned medium was removed with 1x wash. The incubation saline solution consisted of (in mM): 111.31 sodium chloride, 3.33 potassium chloride, 20 sodium bicarbonate, 10 4-(2-hydroxyethyl)-1-piperazineethanesulfonic acid (HEPES), 2 calcium chloride, 1 magnesium chloride, 0.001 2,3-Dioxo-6-nitro-1,2,3,4-tetrahydrobenzo[*f*]quinoxaline-7-sulfonamide (NBQX, Tocris), 0.05 D-(-)-2-Amino-5-phosphonopentanoic acid (D-APV, Tocris). High K^+^ incubation saline was made by equimolar exchange of 30 mM sodium chloride with potassium chloride. Cultures were then placed in a humidified incubator (37°C, 5% CO_2_/95% air) for 16 h. Neuronal survival was then assessed by incubating dishes for 30 minutes with Hoechst 33342 (5 μM) to label all nuclei and propidium iodide (3 μM) to identify neurons with compromised membranes, interpreted as cell death. Ten 20x fields were chosen semi-randomly (must have at least one identifiable viable or non-viable neuron via phase microscopy) for each condition, yielding ~50 cells total per condition. Cell counts were performed using ImageJ (ImageJ Software; NIH, Bethesda, MD) thresholding and counting algorithms. Survival was quantified as the ratio of propidium iodide-negative cells to the total number of neurons (times 100%). Survival experiments were conducted as a dependent sample design; sibling cultures from the same litter were simultaneously seeded and treated, and means of independent replicate experiments were compared by repeated measures statistics.

### Electrophysiology

Whole-cell electrophysiological recordings were performed at room temperature on the stage of an Eclipse TE2000-S inverted microscope. Data were collected using a Multiclamp 700B amplifier and Digidata 1550 data acquisition board (Molecular Devices) using pClamp 10 software. Experiments studying evoked autaptic excitatory postsynaptic currents (EPSCs) used an intracellular pipette solution consisting of (in mM): 140 potassium gluconate, 4 NaCl, 10 HEPES, 5 Ethylene glycol-bis(2-aminoethylether)-*N*,*N*,*N′*,*N′*-tetraacetic acid (EGTA, Sigma), and 0.5 CaCl_2_. The pH was adjusted to 7.25 with KOH. Exogenous ATP, MgATP, and glucose were not added to the whole-cell pipette solution. Following incubation in incubation saline solution for ~16 hours, culture medium was exchanged with an extracellular recording solution for electrophysiological studies. Extracellular solution during voltage-clamp recordings typically consisted of (in mM): 138 NaCl, 4 KCl, 10 HEPES, 2 CaCl_2_, 1 MgCl_2_, and 0.01 D-APV, pH 7.25, adjusted with NaOH.

Whole-cell recording pipettes were pulled from borosilicate glass capillary tubes (World Precision Instruments) and exhibited 2–6 MΩ final open-tip resistances. Unless otherwise stated, neurons in voltage-clamp mode were held at -70 mV. Access resistance was compensated to 90–95% for evoked autaptic PSC recordings. Evoked autaptic PSCs were elicited with a 1.5 ms depolarizing pulse to 0 mV and data were sampled at 20 kHz, filtered at 10 kHz for PSC recordings. Methods to monitor the recovery of EPSCs following vesicle depletion have been described previously [9]. Briefly, after patching a solitary glutamatergic autaptic neuron, autaptic EPSCs were evoked every 25 seconds until a stable baseline was achieved. Neurons were then challenged with 90 mM KCl (equimolar substitution with NaCl) for 30 s to deplete the total recycling vesicle pool. Immediately after high K^+^ application EPSCs were recorded every 25 sec for at least 5 sweeps. The charge transfer of evoked EPSCs at each time point following vesicle depletion was normalized to the charge transfer of the averaged baseline EPSC.

### Glycogen and lactate measurements

Glycogen content of cultures was determined by using a commercial kit (Abcam ab65620) following the manufacturer’s instructions. In brief, cells were rinsed with PBS, homogenized, and centrifuged. Aliquots of supernatant were stored for glycogen and BCA protein assays (Thermo Scientific). Glycogen values were normalized to the amount of protein in each sample.

Lactate concentration in the conditioned saline following prolonged glucose deprivation was measured using the YSI 2900 analyzer (YSI Incorporated, Yellow Springs, OH) as per manufactures directions. Conditioned saline (50 μl) was collected following incubation and stored at -20°C for ~1–2 weeks prior to measurement.

### Data analysis

Data was analyzed and plotted using MetaMorph 7, Clampfit 10 (Molecular Devices), Excel 2011 (Microsoft), Prism 6 (GraphPad), and ImageJ. Unless otherwise stated, data in figures and text are given as mean ± SEM. Student’s two-tailed unpaired *t* test was used to compare 2 groups unless otherwise noted. If more than 2 parameters were compared between 2 groups, a Bonferroni correction was applied unless otherwise noted. A one-way ANOVA was used if comparing more than two experimental conditions and a two-way ANOVA was implemented when comparing the effects of at least two conditions over time or two distinct conditions under at least two treatments. Significance was defined as a corrected p-value < 0.05. The reported n refers to the number of neurons in each group within a particular experiment, except in imaging/neuronal survival experiments where it refers the number of independent culture platings. In all cases at least 3 independent cultures were surveyed, each contributing equally to final N values.

### Materials

D-APV, NBQX, and TTX were obtained from Tocris Biosciences. All materials without identified suppliers above were obtained from Sigma-Aldrich.

## Results

### Hippocampal neurons are resilient to prolonged glucose deprivation

**Fig 1:**
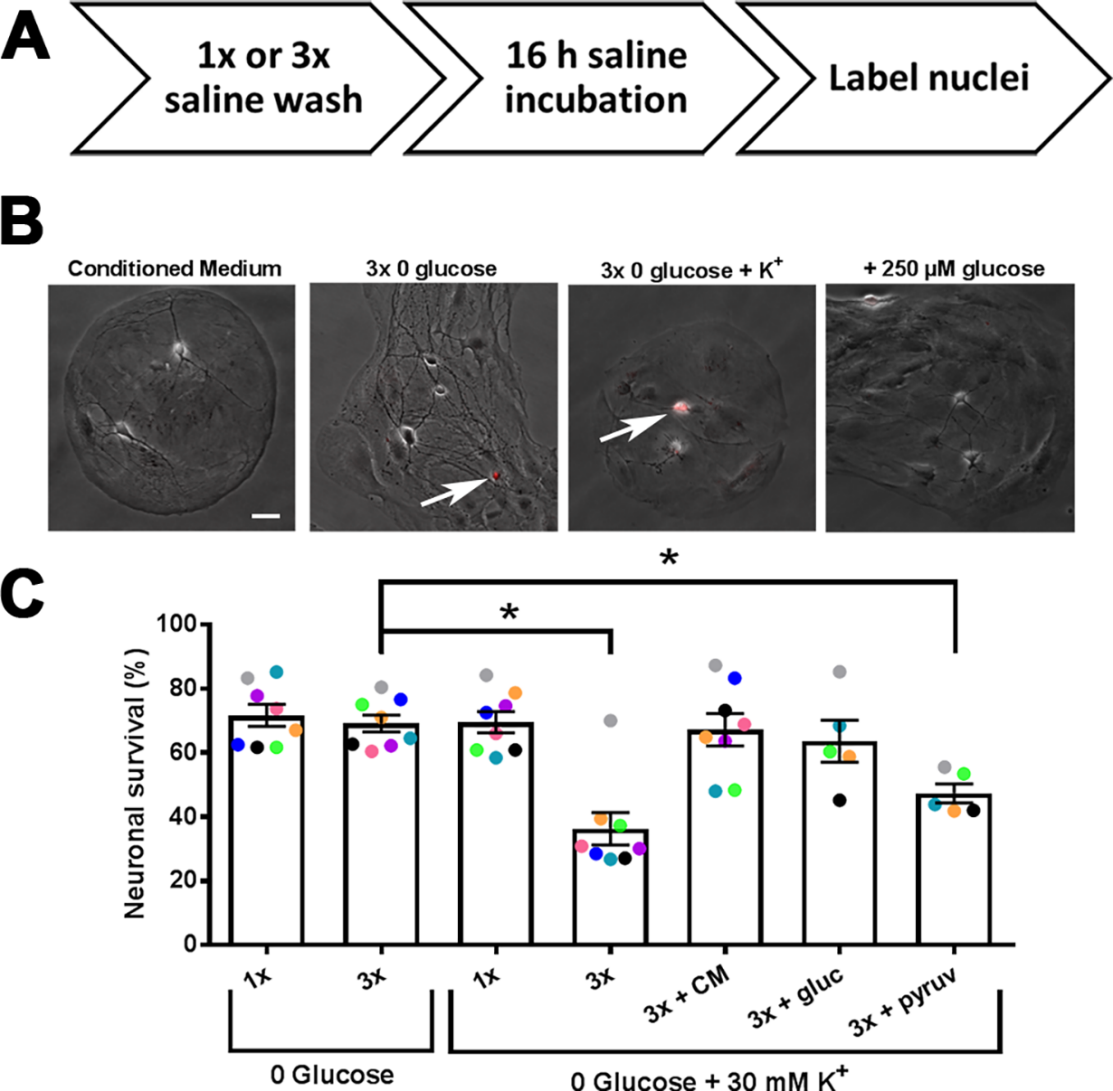
Neuronal viability is resilient to prolonged glucose deprivation. (A) Schematization of survival protocol. Culture medium was either exchanged for glucose-free saline prior to prolonged incubation (1x), or cultures were washed twice in glucose-free saline prior to prolonged incubation in the 3^rd^ application of glucose-free saline for 16 h (3x). In some cases alterations were made to the incubation solution, such as the addition of 30 mM KCl, glucose, etc. Following prolonged incubation, propidium iodide was used to identify nuclei of compromised neurons. (B) Sample phase-contrast images of microcultures overlaid with red fluorescent propidium iodide stain (arrows) following prolonged incubation in conditioned medium, 3x glucose deprivation, 3x glucose deprivation + K^+^, and 3x glucose deprivation + K^+^, 250 μM glucose. Scale bar = 20 μm. (C) Summary of neuronal survival expressed as the percentage of propidium iodide-negative neurons in 10 semi-randomly chosen fields (see Materials and Methods). Symbols are color coded to indicate sibling cultures; different colors represent independent platings. (p<0.05, One-way ANOVA with Dunnett’s multiple comparisons test with 3x glucose deprivation as control). Data are represented as mean ± SEM. *p<0.05.

To test the effect of prolonged glucose deprivation on neuronal survival, we incubated DIV 12–15 rat hippocampal microcultures for 16 h at 37°C in defined, incubation saline solution lacking glucose and trophic factors but containing glutamate receptor blockers to prevent secondary excitotoxicity (see Methods). We deemed this necessary because in pilot experiments on 6 cultures challenged with glucose deprivation with and without glutamate receptor antagonists, we found significant blocker protection in half the experiments. The evidence for variable excitotoxicity prompted us to perform all successive experiments in glutamate receptor antagonists to mitigate excitotoxicity and focus results on core metabolic susceptibility. As schematized in Fig 1A, neuronal cell death was quantified with the fluorescent marker, propidium iodide (Fig 1B, red fluorescence overlay). We were surprised to find that neither a simple exchange of conditioned growth medium with 0-glucose saline (1x) nor multiple washes with 0-glucose saline to remove trace conditioned medium prior to prolonged glucose deprivation (3x) resulted in significant cell death (Fig 1B and 1C). By comparison, survival in cultures with no medium exchanges was 83 ± 6% (n = 3). The results of Fig 1 suggest that hippocampal neuronal survival is quite tolerant of glucose deprivation in the absence of secondary excitotoxicity. As expected, glycogen content in the cultures decreased with overnight glucose deprivation (S1 Fig), likely providing at least a partial explanation for the resilience.

By pharmacologically inhibiting glutamate receptors and circumventing excitotoxicity, we isolated core contributions to neuroenergetic demise. However, this also decreased electrical activity in the networks, which may aid neuroenergetic resilience. Synaptic transmission accounts for the majority of ATP consumption,[1,2] and presynaptic ATP is quickly depleted in an activity-dependent manner[8,28]. We reasoned that neuronal cell loss might be hastened by a concurrent depolarization in the glucose-free saline (0 glucose + 30 mM KCl), which would simulate neuronal electrical activity and stimulate synaptic vesicle cycling, among other energetic demands associated with depolarization[29–31]. Consistent with this idea, we found that 3x medium exchange with glucose-free saline and 30 mM K^+^ produced significant neuronal loss (Fig 1B and 1C). Interestingly, simple medium exchange with depolarizing glucose-free saline resulted in no significant loss (Fig 1C). Addition of a 1:100 dilution of conditioned medium to the 3x depolarizing glucose-free saline condition protected against death (Fig 1C), verifying that cell survival in the 1x condition likely results from trace amounts of medium lingering after the solution exchange. Similar protection was achieved by adding 250 μM glucose (1:100 of that in culture medium) to the 3x depolarizing glucose-free saline, suggesting that death resulted from glucose deprivation rather than from loss of trophic support (Fig 1C).

The other major component of culture medium that could readily serve as a source of ATP production is pyruvate. However, dilute pyruvate (1:100; 0.0022 mM), did not significantly increase survival during depolarizing glucose deprivation, indicating that pyruvate is unlikely to account for the effect of dilute culture medium (Fig 1C). On the other hand, the full pyruvate concentration found in culture medium provided full protection (S2 Fig), consistent with the idea that pyruvate is an effective neuronal energetic substrate. None of the treatments depicted in Fig 1 produced notable propidium iodide staining in astrocytes or consistently altered the morphological appearance of astrocytes (Fig 1B).

It is interesting that a significant fraction of neurons survived following the depolarizing glucose deprivation condition. However, we found in three independent experiments that all neurons and most astrocytes died following 16 h co-incubation in glucose-deprived medium with 1 μM oligomycin to inhibit OXPHOS, regardless of the presence of depolarizing potassium (S3 Fig). Thus, cells are indeed dependent on the combination of glycolysis and OXPHOS during the 16 h period.

### Synaptic transmission is unaffected by prolonged glucose deprivation

**Fig 2:**
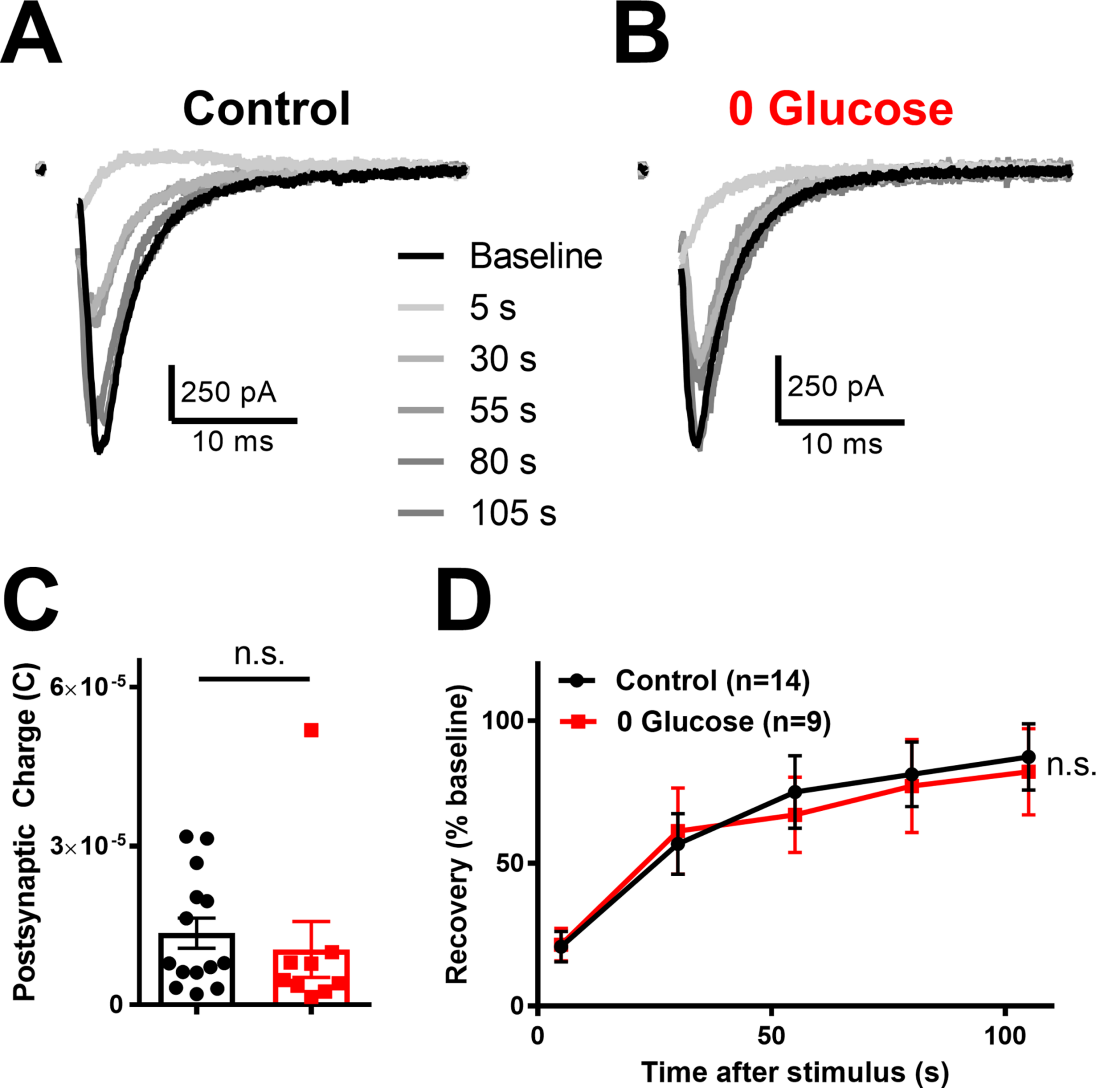
Synaptic transmission is intact following prolonged glucose deprivation. (A,B) Representative baseline action potential evoked EPSCs (black traces) and subsequent EPSC recovery following vesicle depletion (gray traces) following control incubation in conditioned medium (A) and following prolonged glucose deprivation (B). (C) Summary of baseline charge transfer following control incubation (black bar/symbols) or glucose deprivation (red bar/symbols; p > 0.05, Student’s t-test). (D) Summary of recovery EPSCs following vesicle depletion protocol (90 mM KCl applied for 30 sec) for conditioned medium (Control; black trace) compared to glucose deprivation (red trace; p > 0.05; Two-way repeated measures ANOVA). Data are represented as mean ± SEM. n.s, non-significant.

Although neuronal survival was maintained following prolonged glucose deprivation, synaptic transmission may be more sensitive than survival as a result of the outsized ATP consumption of neurotransmission[2,32]. To test tolerance of synaptic transmission to prolonged glucose deprivation compared to glucose-incubated controls, we measured the charge transfer of evoked EPSCs (Fig 2A and 2B black traces). In this experiment and throughout the rest of this report, glucose deprivation is defined as the 3x wash condition applied for 16 h, and recordings were performed in the same glycemic state. Like survival, EPSC charge transfer was unaffected by prolonged glucose deprivation (Fig 2C). In addition, 16 h glucose deprivation did not alter passive properties of neurons (S4 Fig). These data suggest that low-frequency, basal evoked synaptic transmission tolerates prolonged glucose deprivation.

Previously, we found that a more energetically demanding synaptic task is sensitive to inhibition of OXPHOS[9]. During this high-demand task we measured recovery of evoked EPSCs following application of 90 mM KCl for 30 s, a protocol that depletes the total vesicle pool [33,34] and found can be fueled by either neuronal glycolysis or by extracelluarly derived monocarboxylates[9]. In the absence of glucose in the present work, it seems likely that monocarboxylates would be the only remaining fuel source, but it is not clear whether endogenous levels following glucose deprivation can sustain a high-demand task. We found that prolonged glucose deprivation did not alter EPSC recovery after total vesicle depletion compared to sibling controls (Fig 2A and 2B gray traces 2D). These data show both basal and high-demand synaptic transmission are unaffected by prolonged glucose deprivation. Taken together, these results suggest that neuronal OXPHOS may still be intact following prolonged glucose deprivation and reflects another facet of impressive neuronal resilience under stark, controlled conditions.

### Neuronal resilience during glucose deprivation is supported by endogenous monocarboxylate shuttling

Because the composition of the cell culture medium during prolonged glucose deprivation was defined and minimalist, we reasoned that the substrate available for neurons must be provided by an endogenous, cellularly derived fuel source. Prevailing views suggest that astrocyte-derived lactate, perhaps released in response to neuronal activity, is important for neuronal OXPHOS and for sustaining ATP levels [35]. To test whether endogenously released monocarboxylates, such as lactate, account for neuronal resilience in the stark microculture environment, we obstructed monocarboxylate shuttling with the monocarboxylate transporter (MCT) inhibitor, 4-CIN (100 μM), during 16 h glucose deprivation.

**Fig 3:**
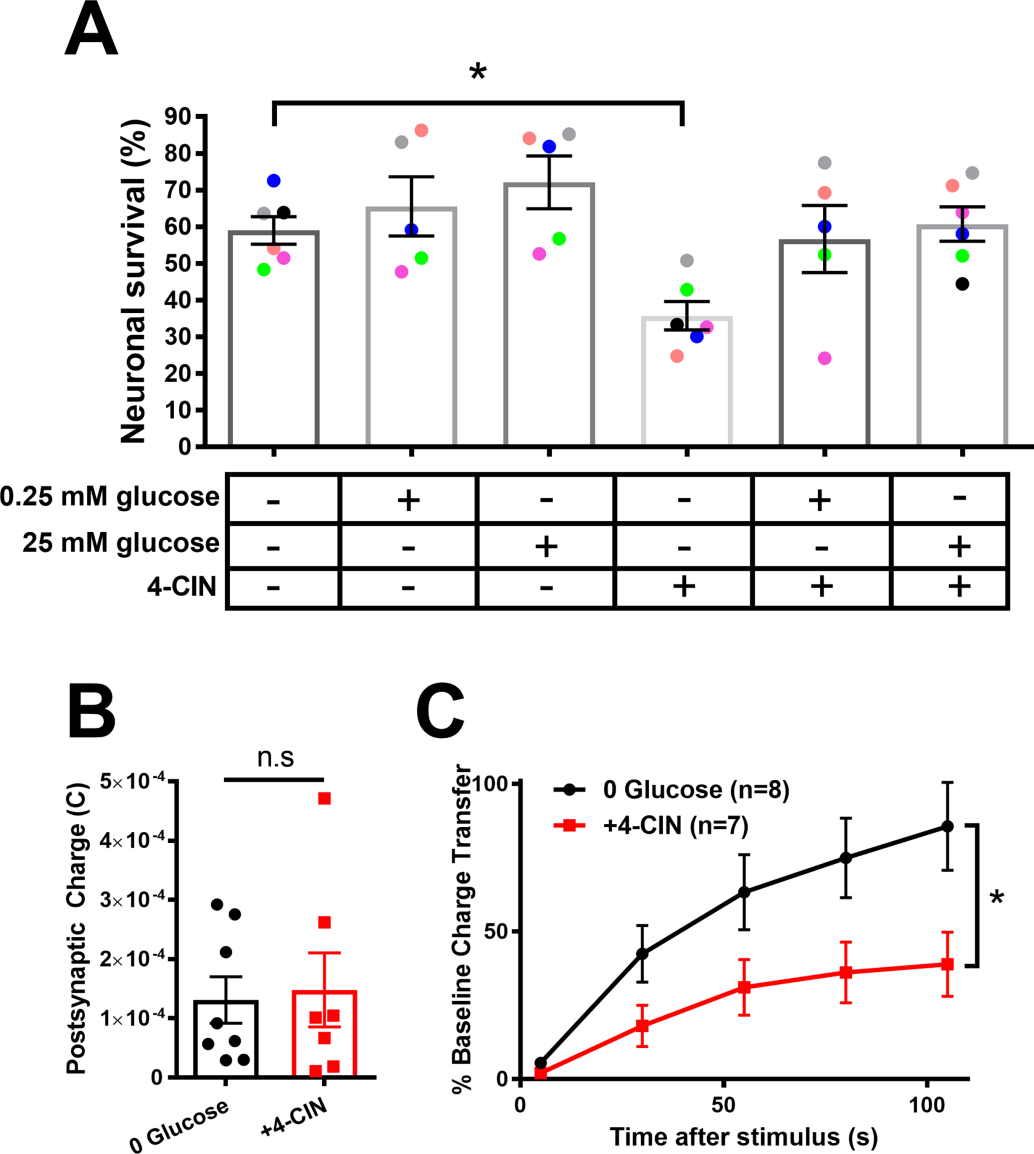
Monocarboxylate shuttling underlies neuronal resilience during prolonged glucose deprivation. (A) Neuronal survival was measured following prolonged incubation in indicated treatment conditions. The ‘+’ and ‘-’ indicate presence and absence, respectively. Data points represent individual cultures, color coded to indicate siblings (one-way ANOVA with Dunnett’s multiple comparisons test). (B) Basal evoked EPSCs are not affected by the absence (black bar/symbols) or presence (red bar/symbols) of monocarboxylate transport inhibitor, 4-CIN (100 μM for 2 h preceding recording), following prolonged glucose deprivation (p > 0.05 Student’s t-test). (C) Cells from B were subjected to K^+^-induced vesicle depletion, as in Fig 2D. EPSCs subjected to glucose deprivation without (black trace) and with 4-CIN (red traces; p<0.05, Two-way repeated measures ANOVA). Summary data are represented as mean ± SEM. *p<0.05. n.s., non-significant.

Co-incubation of neurons in 4-CIN and 0 glucose significantly reduced neuronal survival (Fig 3A). Either trace (0.25 mM) or full (25 mM) glucose preserved neuronal survival in the presence of 4-CIN, suggesting that cell death was unlikely due to off-target effects of the drug (Fig 3A). These data also suggest that cells may be able to utilize even trace glucose to promote neuronal survival, even when monocarboxylate transport is inhibited.

We also tested whether basal and high-demand synaptic transmission were compromised by monocarboxylate transport inhibition (Fig 3B and 3C). We found that 16 h glucose deprivation combined with 2 h monocarboxylate transport inhibition (to avoid cell loss observed in Fig 3A) produced no deficit in basal transmission (Fig 3B) but compromised the ability of evoked EPSCs to recover following K^+^-induced depolarization and vesicle depletion, as described earlier (Fig 3C). These results suggest that neurons utilize monocarboxylate transport to supply OXPHOS needed for neuronal survival and synaptic transmission following prolonged glucose deprivation.

### Local astrocyte support does not affect neuronal resilience to prolonged glucose deprivation

The sensitivity of neurons to monocarboxylate transport inhibition suggests that endogenously released monocarboxylates, perhaps lactate from neighboring astrocytes, may participate in neuroenergetic resilience[35–37]. To test the role of local astrocyte lactate shuttling in neuronal tolerance to glucose deprivation, we utilized our cell culture system which allows us to grow neurons on either a bed of astrocytes (+astrocyte microcultures) or a bed of collagen, void of local astrocyte support (-astrocyte microcultures) within the same cell culture dish and under the same global conditions[27]. We first examined neuronal survival on +astrocyte microcultures compared to -astrocyte microcultures. In pooled results from 11 independent culture platings, we found that survival within the same cell culture dish was 81 ± 4.4% (-astrocyte neurons) vs. 68 ± 5.4% (+astrocyte neurons) at baseline. In 0 glucose, similar results were obtained in glucose deprived cultures (74 ± 4.1% survival in -astrocyte neurons vs. 64 ± 6.7% survival in +astrocyte neuron counterparts). A two-way ANOVA revealed unexpected lower overall survival in +astrocyte microcultures (p = 0.039), in contrast with local astrocyte support hypothesis. The loss was evident in both glucose-containing medium as well with glucose deprivation, suggesting the difference is unrelated to glucose-deprivation. Instead, this astrocyte-associated loss could represent previously characterized astrocyte-associated neuronal apoptosis[38].Taken together, these data suggest that loss of local astrocyte support does not negatively affect neuronal survival following prolonged glucose deprivation.

The astrocyte-neuron lactate shuttle hypothesis predicts that in response to glutamate release from neurons, astrocytes increase glycolysis as a result of the Na^+^-dependent clearance of glutamate from the synaptic cleft, resulting ultimately in lactate production and shuttling to neurons to sustain excitatory neurotransmission[35]. To test the importance of local astrocyte support in sustaining synaptic transmission in the context of glucose deprivation, we compared basal and high-demand synaptic transmission of neurons grown without underlying astrocytes (-astrocyte EPSCs) following prolonged incubation in control medium or glucose-free saline. We previously showed that removal of local astrocytes does not affect either basal or high-demand synaptic transmission when glucose is present[9].

**Fig 4:**
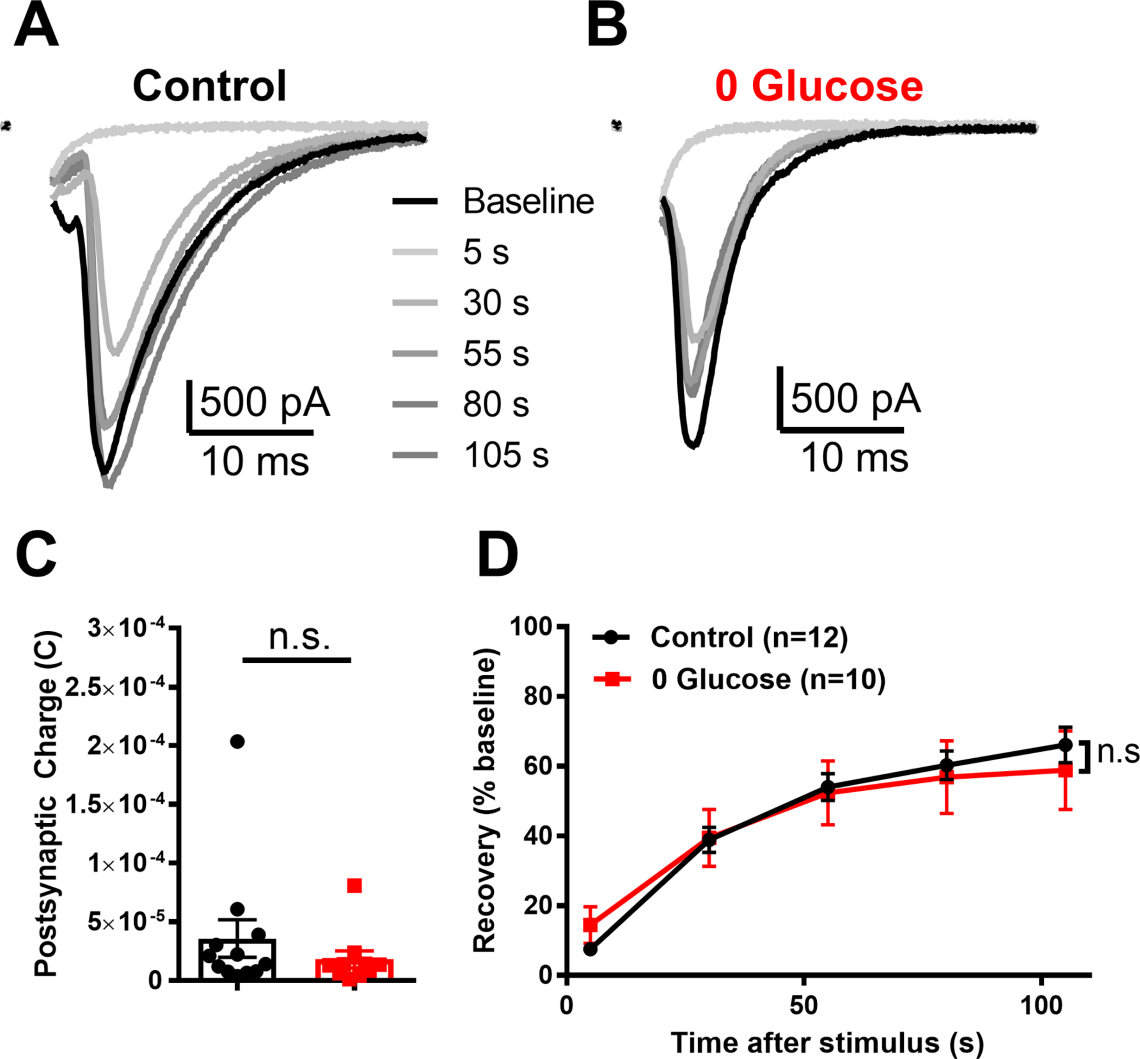
Loss of local astrocyte support does not affect basal or high-demand transmission following prolonged glucose deprivation. (A,B) Representative baseline evoked EPSCs (black traces) and subsequent recovery following K^+^-evoked vesicle depletion (gray traces) of -astrocyte neurons incubated in conditioned medium (A, Control) and following prolonged glucose deprivation (B, 0 Glucose). (C) Summary of baseline charge transfer following prolonged incubation in either conditioned medium (Control; black bar/symbols) or glucose deprivation (red bar/symbols; p > 0.05, Student’s t-test). (D) Summary of recovery of evoked EPSCs following K^+^-induced vesicle depletion for conditioned medium controls (black trace) compared to glucose deprivation (red trace; p > 0.05; Two-way repeated measures ANOVA). Data are represented as mean ± SEM. n.s., non-significant.

Following prolonged glucose deprivation, basal evoked EPSCs from -astrocyte neurons showed no decrement in total postsynaptic charge transfer relative to EPSCs from -astrocyte glutamatergic neurons incubated in control conditions (Fig 4A–4C). Furthermore, glucose deprivation did not alter -astrocyte EPSC recovery following K^+^-mediated vesicle depletion (Fig 4A, 4B and 4D). Because of the previously reported importance of OXPHOS to EPSC recovery, this result suggests that absence of local astrocyte support does not impair OXPHOS enough to reduce transmission during this high-demand task. Taken together, these findings suggest that local, glutamate-stimulated astrocyte shuttling is not responsible for sustaining OXPHOS-dependent presynaptic demand, even following prolonged glucose deprivation. Rather, it is possible that global ambient lactate is important for neuroenergetic resilience of synaptic transmission.

### Increased extracellular lactate mediates neuronal resilience during prolonged glucose deprivation

**Fig 5:**
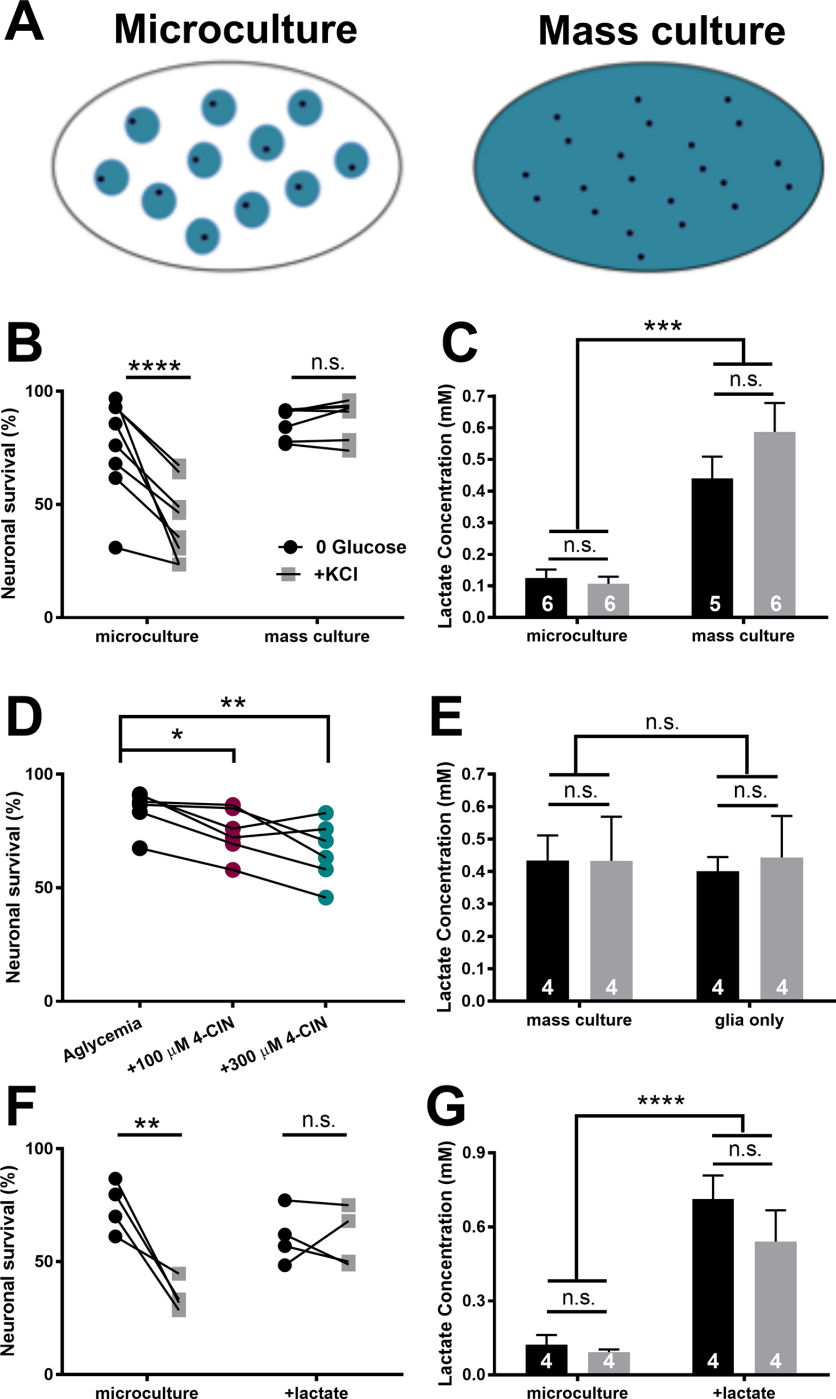
Ongoing, global lactate release supports neuronal resilience to glucose deprivation. (A) Schematic of microcultures and high-density mass cultures. Compared with mass cultures (right), microcultures (left) typically contain fewer neurons (black dots) and fewer astrocytes overall (blue circles), but with similar local density of astrocytes [38]. (B) Survival of microcultures and mass cultures, incubated in either glucose-free saline (0 glucose, black dots) or in glucose-free depolarizing saline (+KCl, gray dots). Lines connect sibling cultures. Two-way ANOVA with repeated measures for depolarization state showed a significant interaction between culture condition and depolarization state (p < 0.001). Results of post-hoc, Bonferroni-corrected multiple comparisons are indicated above symbols. (C) Summary of lactate concentrations measured from microcultures and mass cultures following prolonged incubation in glucose-free saline (black bars) or glucose-free depolarizing saline (gray bars; Two-way ANOVA with no significant difference between glucose deprivation ± KCl and p < 0.001 between microculture and mass culture). (D) 4-CIN induces neuronal loss in mass cultures at both 100 μM (teal dots) and 300 μM (burgundy dots). Lines connect sibling cultures. One-way repeated measures ANOVA with Dunnett’s multiple comparisons test with glucose deprivation as control. (E) Lactate concentration measured in mass cultures in the absence of neurons does not differ from that measured in the presence of neurons. Black bars indicate prolonged glucose deprivation and gray bars indicate prolonged depolarizing glucose deprivation (+KCl; p > 0.05, Two-way ANOVA). (F) Summary of neuronal survival for microcultures incubated in glucose-free saline with or without 0.35 mM lactate added to mimic mass culture lactate. Two-way ANOVA with repeated measures for depolarization state revealed a significant interaction between lactate and depolarization state (p < 0.05). Results of post-hoc Bonferroni corrected multiple comparisons are indicated above symbols. (G) Summary of lactate concentrations measured from microcultures with or without added lactate, confirming persistence of lactate addition (Two-way ANOVA with no significant difference between glucose deprivation ± KCl and p < 0.0001 between microculture ± lactate). Data are represented as mean ± SEM. *p<0.05. **p<0.01. ***p<0.001. ****p<0.0001. n.s., non-significant.

The results above suggest that global monocarboxylate concentration, not local, on-demand shuttling, might participate in neuroenergetic resilience observed under reduced conditions. To study lactate’s potential role in survival, we tested differences in neuronal survival between mass cultures and microcultures, reasoning that lactate levels in conventional mass cultures, which contain larger numbers of astrocytes (schematized in Fig 5A)[38], may exhibit more lactate output. As observed in Fig 1, microculture neuronal survival was sensitive to depolarizing glucose deprivation. However, mass cultures were resistant to the same treatment (Fig 5B). Lactate measurements from the two culture environments revealed that lactate concentrations were significantly higher in mass cultures compared to microcultures. Within each group, lactate concentrations were similar in depolarizing or non-depolarizing saline (Fig 5C). We were surprised that any lactate could be produced and maintained for 16 h in the absence of glucose. Indeed, following 16 h incubation in glucose-containing saline (10 mM), mass cultures exhibited much higher extracellular lactate (2.70 ± 0.14 mM; N = 3 cultures), so lactate levels were compromised following prolonged glucose deprivation, but not abolished. Further, we measured lactate at early time points following the switch from conditioned medium. These results revealed a rapid increase in extracellular lactate but failed to reveal an effect of depolarization on lactate at any time point (S5 Fig).

Neuronal survival was diminished in mass cultures when monocarboxylate transport was inhibited during prolonged glucose deprivation (Fig 5D). However, neuronal death was not as pronounced at either 100 μM or 300 μM 4-CIN in mass cultures compared to microcultures treated with 100 μM 4-CIN (Fig 3). The difference seems likely due to higher levels of lactate and the competitive nature of 4-CIN antagonism. Based on these results, we hypothesized that mass-culture concentrations of lactate are neuroprotective against depolarizing glucose deprivation while concentrations of lactate in microculture cannot sustain resilience.

To verify that the observed lactate originates from astrocytes, we compared lactate levels of astrocyte cultures lacking neurons following incubation in glucose-free saline (Fig 5E, black bars) and depolarizing glucose-free saline (Fig 5E, gray bars) with that found in sibling neuron-astrocyte co-cultures. Astrocyte-only mass cultures and neuron-astrocyte co-cultures were matched for astrocyte numbers (co-cultures: 66.6 ± 19.6 glia per field; astrocyte-only cultures: 77.3 ± 27.4 glia per field; N = 4 cultures). Lactate concentrations observed in astrocyte-only cultures were not significantly different than those measured from co-cultures in both glucose-free and depolarizing glucose-free saline (Fig 5E). These data suggest that extracellular lactate following prolonged incubation in glucose-free saline, with or without concurrent depolarization, originates from astrocytes. As a test of whether protective lactate in mass cultures originates from glutamate-stimulated lactate release, we inhibited glutamate uptake with 50 μM D,L-TBOA. This treatment did not affect neuronal survival (S6 Fig), consistent with the idea that lactate efflux is not ‘on-demand’ under these conditions

To test the hypothesis that ambient lactate differences underlie the differences in resilience between mass cultures and microcultures, we added 0.35 mM lactate to microculture glucose-free saline to match the concentration found in mass cultures. Lactate imparted survival tolerance to microcultures during depolarizing glucose deprivation (Fig 5F and 5G). Taken together, these data suggest that increased concentrations of extracellular lactate released by the global population of astrocytes increases neuronal resilience to prolonged glucose deprivation, even when energetic demands are increased by depolarization.

## Discussion

In this study we investigated the effects of prolonged glucose deprivation on neuronal survival and synaptic physiology in a reduced, controlled environment. After 16 h incubation in glucose-free saline, both neuronal viability and synaptic function were preserved. Depolarization decreased survival during glucose deprivation, but trace amounts of glucose in the incubation saline restored tolerance. Inhibition of monocarboxylate transport, which supplies substrates to the TCA cycle/OXPHOS, also significantly decreased neuronal survival, and trace glucose overcame this effect. Recovery of synaptic transmission following vesicle depletion, a task previously found to be sustained preferentially by OXPHOS[9], remained intact following prolonged glucose deprivation. Interestingly, local lactate shuttling from astrocytes in immediate contact with neurons was not required for resilience of survival or synaptic signaling. Instead, bulk lactate maintained the OXPHOS required for high-demand transmission. Overall, our work demonstrates that ambient lactate can preserve neuronal survival and synaptic transmission even in the absence of glucose.

We previously demonstrated that neurons flexibly source substrates available in the extracellular milieu to maintain presynaptic function[9]. When ATP production is acutely inhibited, synaptic transmission is completely abolished, demonstrating the importance of ongoing ATP production for presynaptic function. Despite the need for ongoing ATP production, here we show that neurons can survive and function many hours in the absence of glucose. We have previously shown that either glycolysis or OXPHOS alone can sustain low-frequency neurotransmission. However recovery of EPSCs after vesicle depletion is sensitive to perturbations in OXPHOS[9]. Our data suggest that OXPHOS can sustain high-demand neurotransmission through endogenous factors produced during glucose-free incubation. Exogenous fuels cannot explain resilience because the incubation medium contained no fuels, including monocarboxylates or amino acids.

Our initial work focused on the importance of lactate mediating neuronal resilience to prolonged glucose deprivation. This was driven by previous studies demonstrating lactate’s release into the extracellular space during synaptic activity[36,39–41] and lactate’s ability to sustain neurotransmission in the absence of glucose[4–6,22,40,42–46]. Although our results show that lactate can affect neuronal viability and function following prolonged glucose deprivation, our work did not directly assess the source of lactate. Astrocytes contain glycogen reserves that are utilized during limited glucose availability (S1 Fig)[4,7,13,47,48]. However these stores are rapidly depleted in intact systems following glucose deprivation[13]. Perhaps the high glucose concentrations found in cell culture media enable astrocytes to store more glycogen or glucose itself, compared to intact systems[49], thus allowing astrocytes to sustain extracellular lactate levels longer. It is also possible that astrocytes utilize other endogenously produced substrates, such as organic acids, nucleosides, amino acids like glutamate[50–52], fatty acids and ketone bodies[23,51,53–55]. Glutamate in particular is an interesting candidate because it can be converted into α-ketogluterate and then metabolized through pyruvate recycling[56,57] and OXPHOS[52,58–60]. These substrates were not constituants of the defined culture medium used to challenge cells in our experiments, but it is conceivable that endogenous supplies participate in sustaining neurons. Future work can test the contribution of these metabolic substrates contributes to the resilience of neuronal survival and function.

Lactate utilization in the brain has been highly debated, but a cornerstone has been the astrocyte-neuron lactate shuttle hypothesis[35]. By this hypothesis, release of glutamate triggers in succession: astrocytic glutamate uptake, intracellular sodium elevation, Na^+^/K^+^ ATPase activity, astrocyte glycolysis, and lactate efflux. Unfortunately, the relevance of the local shuttle is difficult to test directly because of the obstacle of removing local astrocyte support in most experimental preparations. Despite their limitations, microcultures offer perhaps a unique opportunity to compare neurotransmission directly with and without local astrocyte support, while leaving global factors unperturbed[27]. Although microcultures may not recapitulate all of the details of local interactions found *in vivo*, neuronal processes are often enveloped by astrocytes[61], and astrocyte glutamate uptake during synaptic signaling is similar to *in situ* hippocampal preparations[62,63]. These observations suggest that the reduced preparation retains key local interactions that could support local shuttling. Nevertheless, local astrocyte absence had no effect on a high-demand, OXPHOS-dependent presynaptic task (Fig 4). Instead, our results suggest that astrocyte support from unstimulated, global lactate efflux is sufficient to sustain neuronal function during glucose deprivation. Of course, we cannot exclude the possibility that in the more complex, three-dimensional context of the brain, cellular interactions could differ, including more prominent local interactions.

Neuronal viability was compromised by depolarizing glucose deprivation in microcultures but not mass cultures. Increased astrocyte numbers in the mass cultures imparted a higher extracellular lactate concentration than microcultures, a concentration difference of 0.2 mM vs. 0.5 mM (Fig 5D and 5E). By comparison, basal interstitial lactate concentrations in human brain are ~0.6 mM, with activity dependent rises in the range of 0.3–0.9 mM[41,64]. On the other hand basal lactate levels in rodent visual cortex measured electrochemically are <0.1 mM[65]. It is interesting to consider that survival resilience may vary by species and/or by brain region in part due to extracellularly available lactate.

The presence or absence of neurons did not significantly affect ambient lactate (Fig 5E). The lack of a detectable difference in lactate concentration between mass cultures with or without neurons may be due to the absence of postsynaptic receptor activation during prolonged glucose deprivation. Postsynaptic activation adds a large energetic load[1,2], and the absence of this energy sink in our experiments may account for the lack of detectable bulk lactate consumption by neurons. Indeed, our experiments lacked explicit exploration of the role of postsynaptic receptor function in neuroenergetic vulnerability and focused on presynaptic demands[8,28,66,67]. Experimental study of postsynaptic receptor contributions to energetic demand is challenging because of the positive feedback nature of postsynaptic excitation and glutamate release[68–70], but perhaps future studies will consider clever methods to interrupt runaway excitation to probe the energetic cost of postsynaptic receptor activation.

The prolonged glucose deprivation and concurrent depolarization required for vulnerability not only depolarizes neurons but also affects astrocytes. Elevated extracellular [K^+^] rapidly stimulates astrocyte glycolysis[29,39,71–74] which underlies increased lactate release[39,75]. We were therefore surprised to observe no significant change in lactate concentrations in either microcultures or mass cultures following prolonged incubation in depolarizing glucose-free saline compared to non-depolarizing glucose deprivation (Fig 5). The timing of lactate measurements following depolarization could influence the ability to detect a depolarization-related component of lactate efflux. In our study, lactate was typically measured following a 16 h incubation compared to < 1 h[75] or < 20 min[39,73,74,76]. However, measurements of lactate soon after depolarization still failed to achieve significant differences (S5 Fig), so the basis of this discrepancy remains unresolved.

Resilience to depolarizing glucose deprivation and to glucose deprivation + 4-CIN was re-established in microcultures with as little as 250 μM glucose (Figs 1C and 3A). Glucose was effective in the presence of monocarboxylate transport inhibition, suggesting that trace glucose may be directly utilized by neurons. On the other hand, 4-CIN is a low-potency antagonist (Fig 5D). Thus, it is possible that trace glucose, in the presence of a non-saturating 4-CIN concentration, triggers sufficient astrocyte glycolysis and lactate efflux to provide neuroprotection. Regardless of whether direct neuronal utilization or indirect astrocyte participation explains survival resilience in trace glucose, because 250 μM glucose is well below the K_d_ value for even high affinity glucose transporters[77], it appears that very modest glucose uptake is sufficient to support survival resilience.

Finally, we acknowledge that resilience studied in the present work could be influenced by the conditions under which the cells were cultivated. For instance, culture preparations are typically prepared and maintained under high-glucose conditions[78,79]. Developmental glucose levels, or levels of any number of factors present in culture medium, could affect neuroenergetic tolerance. Resilience could also represent a feature of the immature cellular state inherent to cultures. Therefore, resilience may represent what neurons are capable of but not necessarily what they normally do. It should be noted that other *in vitro* preparations generally regarded as ‘intact’ also come with caveats, including metabolic disturbances in brain slices as a result of preparation [80]. The molecular mechanisms identified here, because they demonstrate the capabilities of neurons, are worthy of further exploration, with the aim of imparting facets of this resilience to neurons in more complex contexts in vivo, where neurons may exhibit more vulnerability.

In summary, our results highlight the high tolerance of neuronal survival and presynaptic function when deprived of glucose. We found that glucose-stimulated, local astrocyte lactate shuttling does not account for the resilience of synaptic transmission, however global extracellular lactate supports neuronal viability and signaling.

## Supporting information

S1 Fig Glycogen depletion with glucose derivation. Glycogen measurements were performed from mass cultures by commercial fluorometric assay according to the manufacturer’s instructions (Abcam catalogue number ab65620). Protein content was determined by BCA. Lines connect pairs of sibling cultures. Results showed a significant reduction in glycogen with glucose deprivation (p = 0.03, n = 6, Wilcoxon signed rank test). The inset shows values normalized to the corresponding 10 mM value.
(TIF)

S2 Fig High pyruvate concentration (0.22 mM) provides full neuroprotection. Colors correspond to sibling cultures. One-way ANOVA with Dunnett’s multiple comparisons. Data are represented as mean ± SEM. *p<0.05, n.s non-significant.
(TIF)

S3 Fig Oligomycin added to glucose deprivation devastates neuronal survival. **A.** Survival of neurons following glucose deprivation. **B.** Neurons and most astrocytes were eliminated by addition of 1 μM oligomycin to the incubation solution. Photomicrographs are representative of 3 independent experiments. Scale bar, 25 μm.
(TIF)

S4 Fig Passive properties after glucose deprivation. Experimental conditions were 0 glucose and 10 mM glucose conditions were incubated in defined saline solution containing the indicated glucose for 16 h prior to recording in the same glucose condition. No effect of glucose deprivation was detected on any parameter. Holding current (Hold) was current at -70 mV.
(TIF)

S5 Fig Extracellular lactate measurements at early time points following glucose deprivation in mass cultures. (TIF)

S6 Fig Effect of 50 μM D,L-TBOA on survival of mass culture neurons in 4 independent experiments. Incubations in all treatments were for 16 h. K^+^ concentration was 30 mM.
(TIF)

S1 Data Supporting information. Data for all figures and supplemental figures.
(XLSX)

## References

[1] Attwell D, Laughlin SB. An energy budget for signaling in the grey matter of the brain. J Cereb Blood Flow Metab. 2001;21: 1133–45. 11598490 10.1097/00004647-200110000-00001

[2] Harris JJ, Jolivet R, Attwell D. Synaptic energy use and supply. Neuron. 2012;75: 762–77. doi: 10.1016/j.neuron.2012.08.019 22958818 10.1016/j.neuron.2012.08.019

[3] Lipton P, Whittingham TS. Reduced ATP concentration as a basis for synaptic transmission failure during hypoxia in the in vitro guinea-pig hippocampus. J Physiol. 1982;325: 51–65. 6286944 10.1113/jphysiol.1982.sp014135 PMC1251379

[4] Brown AM, Sickmann HM, Fosgerau K, Lund TM, Schousboe A, Waagepetersen HS, et al Astrocyte glycogen metabolism is required for neural activity during aglycemia or intense stimulation in mouse white matter. J Neurosci Res. 2005;79: 74–80. doi: 10.1002/jnr.20335 15578727 10.1002/jnr.20335

[5] Schurr A, West CA, Rigor BM. Lactate-supported synaptic function in the rat hippocampal slice preparation. Science. 1988;240: 1326–8. 3375817 10.1126/science.3375817

[6] Izumi Y, Benz AM, Zorumski CF, Olney JW. Effects of lactate and pyruvate on glucose deprivation in rat hippocampal slices. Neuroreport. 1994;5: 617–20. 8025256 10.1097/00001756-199401000-00021

[7] Wender R, Brown AM, Fern R, Swanson RA, Farrell K, Ransom BR. Astrocytic glycogen influences axon function and survival during glucose deprivation in central white matter. J Neurosci. 2000;20: 6804–10. 10995824 10.1523/JNEUROSCI.20-18-06804.2000 PMC6772835

[8] Rangaraju V, Calloway N, Ryan TA. Activity-driven local ATP synthesis is required for synaptic function. Cell. 2014;156: 825–835. doi: 10.1016/j.cell.2013.12.042 24529383 10.1016/j.cell.2013.12.042 PMC3955179

[9] Sobieski C, Fitzpatrick MJ, Mennerick S. Differential presynaptic ATP supply for basal and high-demand transmission. J Neurosci. 2017;37: 1888–1899. doi: 10.1523/JNEUROSCI.2712-16.2017 28093477 10.1523/JNEUROSCI.2712-16.2017 PMC5320616

[10] Rich L, Brown AM. Glycogen: Multiple roles in the CNS. Neurosci. 2017;23: 356–363. doi: 10.1177/1073858416672622 27707995 10.1177/1073858416672622

[11] Saez I, Duran J, Sinadinos C, Beltran A, Yanes O, Tevy MF, et al Neurons have an active glycogen metabolism that contributes to tolerance to hypoxia. J Cereb Blood Flow Metab. 2014;34: 945–955. doi: 10.1038/jcbfm.2014.33 24569689 10.1038/jcbfm.2014.33 PMC4050236

[12] Cataldo AM, Broadwell RD. Cytochemical identification of cerebral glycogen and glucose-6-phosphatase activity under normal and experimental conditions: I. Neurons and glia. J Electron Microsc Tech. 1986;3: 413–437. 10.1007/BF01611733 3018177

[13] Brown AM. Brain glycogen re-awakened. J Neurochem. 2004;89: 537–52. doi: 10.1111/j.1471-4159.2004.02421.x 15086511 10.1111/j.1471-4159.2004.02421.x

[14] Rau TF, Lu Q, Sharma S, Sun X, Leary G, Beckman ML, et al Oxygen glucose deprivation in rat hippocampal slice cultures results in alterations in carnitine homeostasis and mitochondrial dysfunction. Ceña V, editor. PLoS One. 2012;7: e40881 doi: 10.1371/journal.pone.0040881 22984394 10.1371/journal.pone.0040881 PMC3439445

[15] Mattson MP, Zhu H, Yu J, Kindy MS. Presenilin-1 mutation increases neuronal vulnerability to focal ischemia in vivo and to hypoxia and glucose deprivation in cell culture: involvement of perturbed calcium homeostasis. J Neurosci. 2000;20: 1358–64. 10662826 10.1523/JNEUROSCI.20-04-01358.2000 PMC6772370

[16] Tombaugh GC, Sapolsky RM. Mild acidosis protects hippocampal neurons from injury induced by oxygen and glucose deprivation. Brain Res. 1990;506: 343–345. 2154291 10.1016/0006-8993(90)91277-n

[17] Wieloch T, Engelsen B, Westerberg E, Auer R. Lesions of the glutamatergic cortico-striatal projections in the rat ameliorate hypoglycemic brain damage in the striatum. Neurosci Lett. 1985;58: 25–30. 2864666 10.1016/0304-3940(85)90323-4

[18] Auer R, Kalimo H, Olsson Y, Wieloch T. The dentate gyrus in hypoglycemia: pathology implicating excitotoxin-mediated neuronal necrosis. Acta Neuropathol. 1985;67: 279–88. 4050343 10.1007/BF00687813

[19] Suh SW, Aoyama K, Chen Y, Garnier P, Matsumori Y, Gum E, et al Hypoglycemic neuronal death and cognitive impairment are prevented by poly(ADP-ribose) polymerase inhibitors administered after hypoglycemia. J Neurosci. 2003;23: 10681–90. 14627653 10.1523/JNEUROSCI.23-33-10681.2003 PMC6740913

[20] Goldberg MP, Choi DW. Combined oxygen and glucose deprivation in cortical cell culture: calcium-dependent and calcium-independent mechanisms of neuronal injury. J Neurosci. 1993;13: 3510–24. 8101871 10.1523/JNEUROSCI.13-08-03510.1993 PMC6576549

[21] Llorente-Folch I, Rueda CB, Perez-Liebana I, Satrustegui J, Pardo B. L-Lactate-mediated neuroprotection against glutamate-induced excitotoxicity requires ARALAR/AGC1. J Neurosci. 2016;36: 4443–4456. doi: 10.1523/JNEUROSCI.3691-15.2016 27098689 10.1523/JNEUROSCI.3691-15.2016 PMC6601833

[22] Izumi Y, Benz AM, Katsuki H, Zorumski CF. Endogenous monocarboxylates sustain hippocampal synaptic function and morphological integrity during energy deprivation. J Neurosci. 1997;17: 9448–57. 9391000 10.1523/JNEUROSCI.17-24-09448.1997 PMC6573429

[23] Izumi Y, Ishii K, Katsuki H, Benz AM, Zorumski CF. beta-Hydroxybutyrate fuels synaptic function during development. Histological and physiological evidence in rat hippocampal slices. J Clin Invest. 1998;101: 1121–32. doi: 10.1172/JCI1009 9486983 10.1172/JCI1009 PMC508664

[24] Nagase M, Takahashi Y, Watabe AM, Kubo Y, Kato F. On-site energy supply at synapses through monocarboxylate transporters maintains excitatory synaptic transmission. J Neurosci. 2014;34: 2605–17. doi: 10.1523/JNEUROSCI.4687-12.2014 24523550 10.1523/JNEUROSCI.4687-12.2014 PMC6802746

[25] Mennerick S, Que J, Benz A, Zorumski CF. Passive and synaptic properties of hippocampal neurons grown in microcultures and in mass cultures. J Neurophysiol. 1995;73: 320–32. doi: 10.1152/jn.1995.73.1.320 7714575 10.1152/jn.1995.73.1.320

[26] Moulder KL, Jiang X, Taylor AA, Shin W, Gillis KD, Mennerick S. Vesicle pool heterogeneity at hippocampal glutamate and GABA synapses. J Neurosci. 2007;27: 9846–54. doi: 10.1523/JNEUROSCI.2803-07.2007 17855599 10.1523/JNEUROSCI.2803-07.2007 PMC6672647

[27] Sobieski C, Jiang X, Crawford DC, Mennerick S. Loss of local astrocyte support disrupts action potential propagation and glutamate release synchrony from unmyelinated hippocampal axon terminals in vitro. J Neurosci. 2015;35: 11105–17. doi: 10.1523/JNEUROSCI.1289-15.2015 26245971 10.1523/JNEUROSCI.1289-15.2015 PMC4524979

[28] Pathak D, Shields LY, Mendelsohn BA, Haddad D, Lin W, Gerencser AA, et al The role of mitochondrially derived ATP in synaptic vesicle recycling. J Biol Chem. 2015;290: 22325–22336. doi: 10.1074/jbc.M115.656405 26126824 10.1074/jbc.M115.656405 PMC4566209

[29] Peng L, Zhang X, Leif H. High extracellular potassium concentrations stimulate oxidative metabolism in a glutamatergic neuronal culture and glycolysis in cultured astrocytes but have no stimulatory effect in a GABAergic neuronal culture. Brain Res. 1994;663: 168–172. 7850466 10.1016/0006-8993(94)90475-8

[30] Ceccarelli B, Grohovaz F, Hurlbut WP. Freeze-fracture studies of frog neuromuscular junctions during intense release of neurotransmitter. II. Effects of electrical stimulation and high potassium. J Cell Biol. 1979;81: 178–92. 39080 10.1083/jcb.81.1.178 PMC2111526

[31] Honegger P, Pardo B. Separate neuronal and glial Na+,K+-ATPase isoforms regulate glucose utilization in response to membrane depolarization and elevated extracellular potassium. J Cereb Blood Flow Metab. 1999;19: 1051–9. doi: 10.1097/00004647-199909000-00013 10478657 10.1097/00004647-199909000-00013

[32] Howarth C, Peppiatt-Wildman CM, Attwell D. The energy use associated with neural computation in the cerebellum. J Cereb Blood Flow Metab. 2010;30: 403–14. doi: 10.1038/jcbfm.2009.231 19888288 10.1038/jcbfm.2009.231 PMC2859342

[33] Sara Y, Mozhayeva MG, Liu X, Kavalali ET. Fast vesicle recycling supports neurotransmission during sustained stimulation at hippocampal synapses. J Neurosci. 2002;22: 1608–17. 11880491 10.1523/JNEUROSCI.22-05-01608.2002 PMC6758906

[34] Chung C, Barylko B, Leitz J, Liu X, Kavalali ET. Acute dynamin inhibition dissects synaptic vesicle recycling pathways that drive spontaneous and evoked neurotransmission. J Neurosci. 2010;30: 1363–76. doi: 10.1523/JNEUROSCI.3427-09.2010 20107062 10.1523/JNEUROSCI.3427-09.2010 PMC2823378

[35] Pellerin L, Magistretti PJ. Sweet sixteen for ANLS. J Cereb Blood Flow Metab. 2012;32: 1152–66. doi: 10.1038/jcbfm.2011.149 22027938 10.1038/jcbfm.2011.149 PMC3390819

[36] Pellerin L, Magistretti PJ. Glutamate uptake into astrocytes stimulates aerobic glycolysis: a mechanism coupling neuronal activity to glucose utilization. Proc Natl Acad Sci U S A. 1994;91: 10625–9. 7938003 10.1073/pnas.91.22.10625 PMC45074

[37] Cater HL, Benham CD, Sundstrom LE. Neuroprotective role of monocarboxylate transport during glucose deprivation in slice cultures of rat hippocampus. J Physiol. 2001;531: 459–66. doi: 10.1111/j.1469-7793.2001.0459i.x 11230518 10.1111/j.1469-7793.2001.0459i.x PMC2278461

[38] Shute AA, Cormier RJ, Moulder KL, Benz A, Isenberg KE, Zorumski CF, et al Astrocytes exert a pro-apoptotic effect on neurons in postnatal hippocampal cultures. Neuroscience. 2005;131: 349–358. doi: 10.1016/j.neuroscience.2004.11.025 15708478 10.1016/j.neuroscience.2004.11.025

[39] Sotelo-Hitschfeld T, Niemeyer MI, Mächler P, Ruminot I, Lerchundi R, Wyss MT, et al Channel-mediated lactate release by K+-stimulated astrocytes. J Neurosci. 2015;35: 4168–4178. doi: 10.1523/JNEUROSCI.5036-14.2015 25762664 10.1523/JNEUROSCI.5036-14.2015 PMC6605297

[40] Schurr A, Miller JJ, Payne RS, Rigor BM. An increase in lactate output by brain tissue serves to meet the energy needs of glutamate-activated neurons. J Neurosci. 1999;19: 34–9. 9870935 10.1523/JNEUROSCI.19-01-00034.1999 PMC6782362

[41] Prichard J, Rothman D, Novotny E, Petroff O, Kuwabara T, Avison M, et al Lactate rise detected by 1H NMR in human visual cortex during physiologic stimulation. Proc Natl Acad Sci U S A. 1991;88: 5829–31. 2062861 10.1073/pnas.88.13.5829 PMC51971

[42] Tang F, Lane S, Korsak A, Paton JFR, Gourine A V., Kasparov S, et al Lactate-mediated glia-neuronal signalling in the mammalian brain. Nat Commun. 2014;5: ra26 doi: 10.1038/ncomms4284 24518663 10.1038/ncomms4284 PMC3926012

[43] Schurr A, Payne RS, Tseng MT, Gozal E, Gozal D. Excitotoxic preconditioning elicited by both glutamate and hypoxia and abolished by lactate transport inhibition in rat hippocampal slices. Neurosci Lett. 2001;307: 151–154. 11438386 10.1016/s0304-3940(01)01937-1

[44] Maus M, Marin P, Israël M, Glowinski J, Prémont J. Pyruvate and lactate protect striatal neurons against N-methyl- d -aspartate-induced neurotoxicity. Eur J Neurosci. 1999;11: 3215–3224. 10510185 10.1046/j.1460-9568.1999.00745.x

[45] Wyss MT, Jolivet R, Buck A, Magistretti PJ, Weber B. In vivo evidence for lactate as a neuronal energy source. J Neurosci. 2011;31: 7477–7485. doi: 10.1523/JNEUROSCI.0415-11.2011 21593331 10.1523/JNEUROSCI.0415-11.2011 PMC6622597

[46] Rouach N, Koulakoff A, Abudara V, Willecke K, Giaume C. Astroglial metabolic networks sustain hippocampal synaptic transmission. Science. 2008;322: 1551–5. doi: 10.1126/science.1164022 19056987 10.1126/science.1164022

[47] Brown AM, Ransom BR. Astrocyte glycogen and brain energy metabolism. Glia. 2007;55: 1263–1271. doi: 10.1002/glia.20557 17659525 10.1002/glia.20557

[48] Dringen R, Gebhardt R, Hamprecht B. Glycogen in astrocytes: possible function as lactate supply for neighboring cells. Brain Res. 1993;623: 208–214. 8221102 10.1016/0006-8993(93)91429-v

[49] Brown AM, Tekkök SB, Ransom BR. Glycogen regulation and functional role in mouse white matter. J Physiol. 2003;549: 501–12. doi: 10.1113/jphysiol.2003.042416 12679378 10.1113/jphysiol.2003.042416 PMC2342948

[50] McKenna MC. The glutamate-glutamine cycle is not stoichiometric: Fates of glutamate in brain. J Neurosci Res. 2007;85: 3347–3358. doi: 10.1002/jnr.21444 17847118 10.1002/jnr.21444

[51] McKenna MC. Substrate competition studies demonstrate oxidative metabolism of glucose, glutamate, glutamine, lactate and 3-hydroxybutyrate in cortical astrocytes from rat brain. Neurochem Res. 2012;37: 2613–26. doi: 10.1007/s11064-012-0901-3 23079895 10.1007/s11064-012-0901-3 PMC3547390

[52] Kreft M, Bak LK, Waagepetersen HS, Schousboe A. Aspects of astrocyte energy metabolism, amino acid neurotransmitter homoeostasis and metabolic compartmentation. ASN Neuro. 2012;4 doi: 10.1042/AN20120007 22435484 10.1042/AN20120007 PMC3338196

[53] Auestad N, Korsak RA, Morrow JW, Edmond J. Fatty acid oxidation and ketogenesis by astrocytes in primary culture. J Neurochem. 1991;56: 1376–1386. 2002348 10.1111/j.1471-4159.1991.tb11435.x

[54] Edmond J, Robbins RA, Bergstrom JD, Cole RA, de Vellis J. Capacity for substrate utilization in oxidative metabolism by neurons, astrocytes, and oligodendrocytes from developing brain in primary culture. J Neurosci Res. 1987;18: 551–61. doi: 10.1002/jnr.490180407 3481403 10.1002/jnr.490180407

[55] Takahashi S, Iizumi T, Mashima K, Abe T, Suzuki N. Roles and regulation of ketogenesis in cultured astroglia and neurons under hypoxia and hypoglycemia. ASN Neuro. 2014;6 doi: 10.1177/1759091414550997 25290061 10.1177/1759091414550997 PMC4187005

[56] Sonnewald U, White LR, Ødegård E, Westergaard N, Bakken IJ, Aasly J, et al MRS study of glutamate metabolism in cultured neurons/glia. Neurochem Res. 1996;21: 987–993. 8897461 10.1007/BF02532408

[57] Waagepetersen HS, Qu H, Hertz L, Sonnewald U, Schousboe A. Demonstration of pyruvate recycling in primary cultures of neocortical astrocytes but not in neurons. Neurochem Res. 2002;27: 1431–7. 12512946 10.1023/a:1021636102735

[58] Hertz L, Drejer J, Schousboe A. Energy metabolism in glutamatergic neurons, GABAergic neurons and astrocytes in primary cultures. Neurochem Res. 1988;13: 605–10. 2901049 10.1007/BF00973275

[59] Swanson RA, Yu AC, Chan PH, Sharp FR. Glutamate increases glycogen content and reduces glucose utilization in primary astrocyte culture. J Neurochem. 1990;54: 490–6. 1967630 10.1111/j.1471-4159.1990.tb01898.x

[60] Dienel GA, Cruz NF. Astrocyte activation in working brain: Energy supplied by minor substrates. Neurochem Int. 2006;48: 586–595. doi: 10.1016/j.neuint.2006.01.004 16513214 10.1016/j.neuint.2006.01.004

[61] Mennerick S, Benz A, Zorumski CF. Components of glial responses to exogenous and synaptic glutamate in rat hippocampal microcultures. J Neurosci. 1996;16: 55–64. 8613809 10.1523/JNEUROSCI.16-01-00055.1996 PMC6578726

[62] Bergles DE, Jahr CE. Synaptic activation of glutamate transporters in hippocampal astrocytes. Neuron. 1997;19: 1297–308. 9427252 10.1016/s0896-6273(00)80420-1

[63] Mennerick S, Zorumski CF. Glial contributions to excitatory neurotransmission in cultured hippocampal cells. Nature. 1994;368: 59–62. doi: 10.1038/368059a0 7906399 10.1038/368059a0

[64] Wijnen JP, Haarsma J, Boer VO, Luijten PR, van der Stigchel S, Neggers SFW, et al Detection of lactate in the striatum without contamination of macromolecules by J-difference editing MRS at 7 T. NMR Biomed. 2015;28: 514–522. doi: 10.1002/nbm.3278 25802216 10.1002/nbm.3278

[65] Li B, Freeman RD. Neurometabolic coupling between neural activity, glucose, and lactate in activated visual cortex. J Neurochem. 2015;135: 742–754. doi: 10.1111/jnc.13143 25930947 10.1111/jnc.13143 PMC4627899

[66] Jang S, Nelson JC, Bend EG, Rodríguez-Laureano L, Tueros FG, Cartagenova L, et al Glycolytic enzymes localize to synapses under energy stress to support synaptic function. Neuron. 2016;90: 278–291. doi: 10.1016/j.neuron.2016.03.011 27068791 10.1016/j.neuron.2016.03.011 PMC4840048

[67] Lujan B, Kushmerick C, Das Banerjee T, Dagda RK, Renden R. Glycolysis selectively shapes the presynaptic action potential waveform. J Neurophysiol. 2016;116: 2523–2540. doi: 10.1152/jn.00629.2016 27605535 10.1152/jn.00629.2016 PMC5133309

[68] Budd SL, Nicholls DG. Mitochondria, calcium regulation, and acute glutamate excitotoxicity in cultured cerebellar granule cells. J Neurochem. 1996;67: 2282–91. 8931459 10.1046/j.1471-4159.1996.67062282.x

[69] Choi DW. Glutamate receptors and the induction of excitotoxic neuronal death. Prog Brain Res. 1994;100: 47–51. 7938533 10.1016/s0079-6123(08)60767-0

[70] Rothman SM, Olney JW. Excitotoxicity and the NMDA receptor—still lethal after eight years. Trends Neurosci. 1995;18: 57–58. 7537407 10.1016/0166-2236(95)93869-y

[71] Bittner XC, Loaiza A, Ruminot I, Larenas V, Barros LF. High resolution measurement of the glycolytic rate. Front Neuroenergetics. 2010;2: 26 doi: 10.3389/fnene.2010.00026 20890447 10.3389/fnene.2010.00026 PMC2947927

[72] Hassel B, Sonnewald U. Effects of potassium and glutamine on metabolism of glucose in astrocytes. Neurochem Res. 2002;27: 167–171. 11926271 10.1023/a:1014827327690

[73] Bittner CX, Valdebenito R, Ruminot I, Loaiza A, Larenas V, Sotelo-Hitschfeld T, et al Fast and reversible stimulation of astrocytic glycolysis by K+ and a delayed and persistent effect of glutamate. J Neurosci. 2011;31: 4709–13. doi: 10.1523/JNEUROSCI.5311-10.2011 21430169 10.1523/JNEUROSCI.5311-10.2011 PMC6622916

[74] Choi HB, Gordon GRJ, Zhou N, Tai C, Rungta RL, Martinez J, et al Metabolic communication between astrocytes and neurons via bicarbonate-responsive soluble adenylyl cyclase. Neuron. 2012;75: 1094–104. doi: 10.1016/j.neuron.2012.08.032 22998876 10.1016/j.neuron.2012.08.032 PMC3630998

[75] Walz W, Mukerji S. Lactate release from cultured astrocytes and neurons: A comparison. Glia. 1988;1: 366–370. doi: 10.1002/glia.440010603 2976396 10.1002/glia.440010603

[76] Ruminot I, Gutiérrez R, Peña-Münzenmayer G, Añazco C, Sotelo-Hitschfeld T, Lerchundi R, et al NBCe1 mediates the acute stimulation of astrocytic glycolysis by extracellular K+. J Neurosci. 2011;31: 14264–14271. doi: 10.1523/JNEUROSCI.2310-11.2011 21976511 10.1523/JNEUROSCI.2310-11.2011 PMC3200293

[77] Thorens B, Mueckler M. Glucose transporters in the 21st Century. Am J Physiol Endocrinol Metab. 2010;298: E141–5. doi: 10.1152/ajpendo.00712.2009 20009031 10.1152/ajpendo.00712.2009 PMC2822486

[78] Kleman AM, Yuan JY, Aja S, Ronnett G V, Landree LE. Physiological glucose is critical for optimized neuronal viability and AMPK responsiveness in vitro. J Neurosci Methods. 2008;167: 292–301. doi: 10.1016/j.jneumeth.2007.08.028 17936912 10.1016/j.jneumeth.2007.08.028 PMC2257477

[79] Bardy C, van den Hurk M, Eames T, Marchand C, Hernandez R V, Kellogg M, et al Neuronal medium that supports basic synaptic functions and activity of human neurons in vitro. Proc Natl Acad Sci U S A. 2015;112: E2725–34. doi: 10.1073/pnas.1504393112 25870293 10.1073/pnas.1504393112 PMC4443325

[80] Takano T, He W, Han X, Wang F, Xu Q, Wang X, et al Rapid manifestation of reactive astrogliosis in acute hippocampal brain slices. Glia. 2014;62: 78–95. doi: 10.1002/glia.22588 24272704 10.1002/glia.22588 PMC4097059

