# Supplementary figures and images for: Ambient but not local lactate underlies neuronal tolerance to prolonged glucose deprivation
Source: PLoS One. 2018 Apr 4;13(4):e0195520. doi: 10.1371/journal.pone.0195520 (PMC5884621; doi:10.1371/journal.pone.0195520)

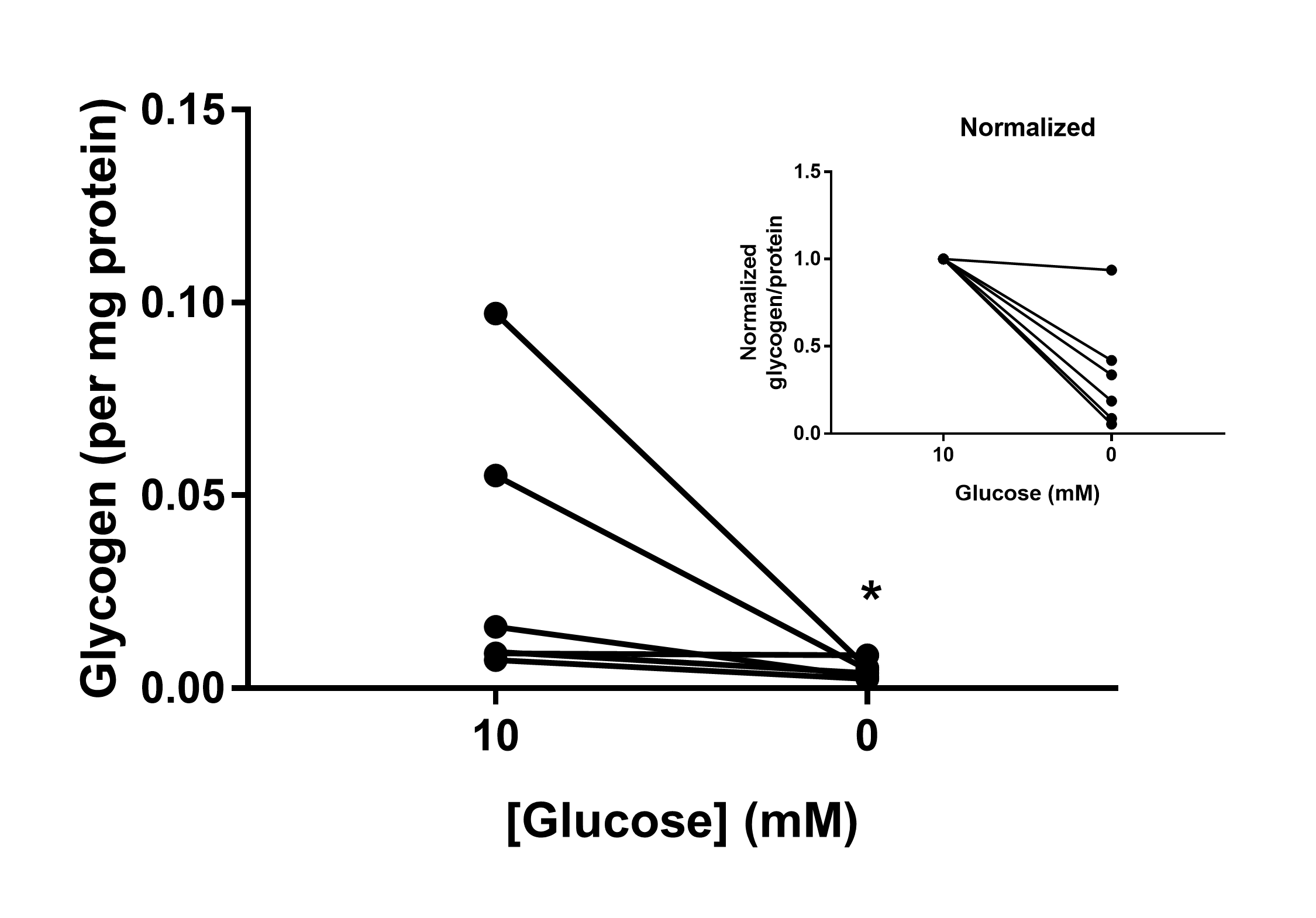

Supplement: S1 Fig — Glycogen measurements were performed from mass cultures by commercial fluorometric assay according to the manufacturer’s instructions (Abcam catalogue number ab65620). Protein content was determined by BCA. Lines connect pairs of sibling cultures. Results showed a significant reduction in glycogen with glucose deprivation (p = 0.03, n = 6, Wilcoxon signed rank test). The inset shows values normalized to the corresponding 10 mM value. (TIF) [file pone.0195520.s001.tif]

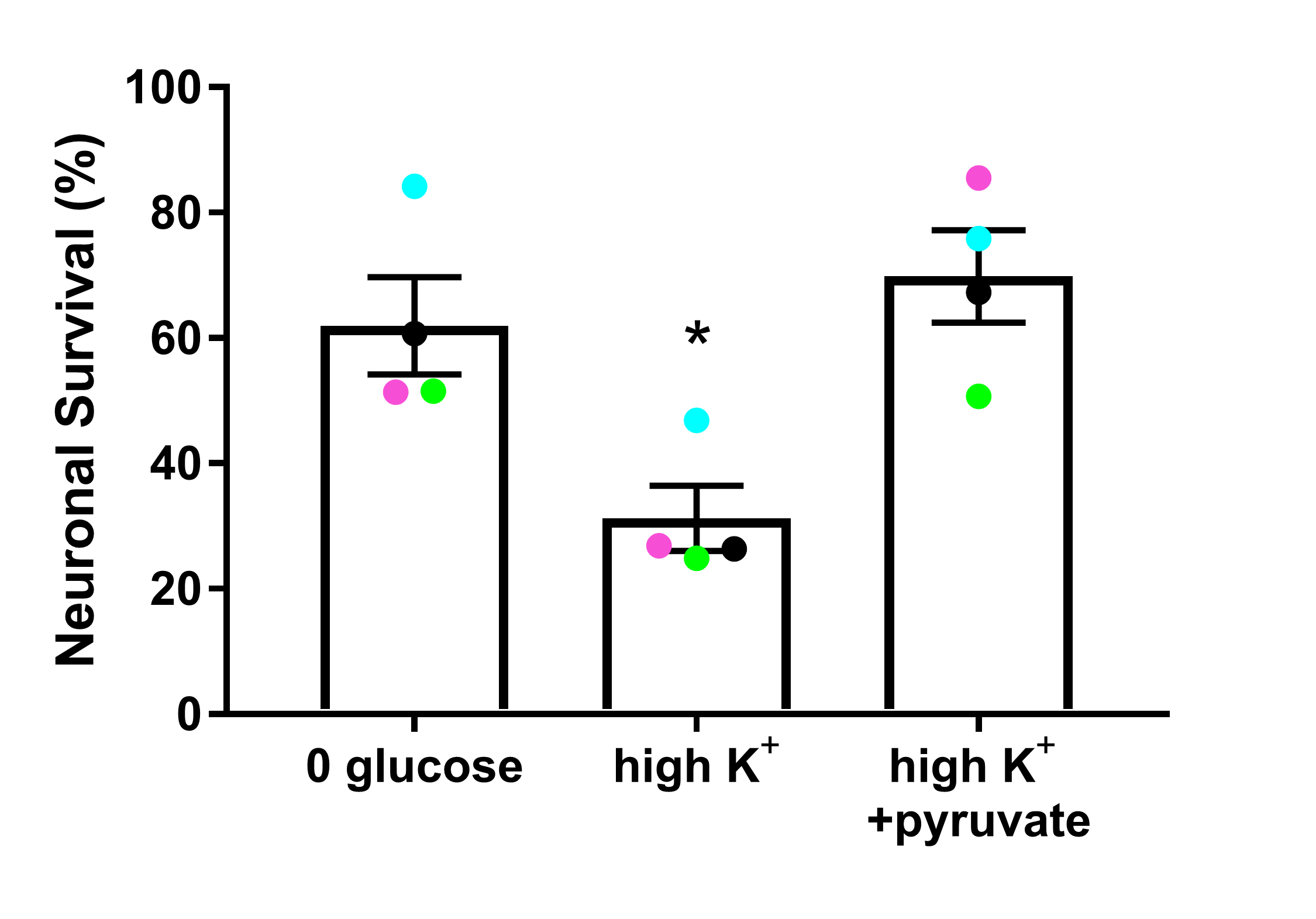

Supplement: S2 Fig — Colors correspond to sibling cultures. One-way ANOVA with Dunnett’s multiple comparisons. Data are represented as mean ± SEM. *p<0.05, n.s non-significant. (TIF) [file pone.0195520.s002.tif]

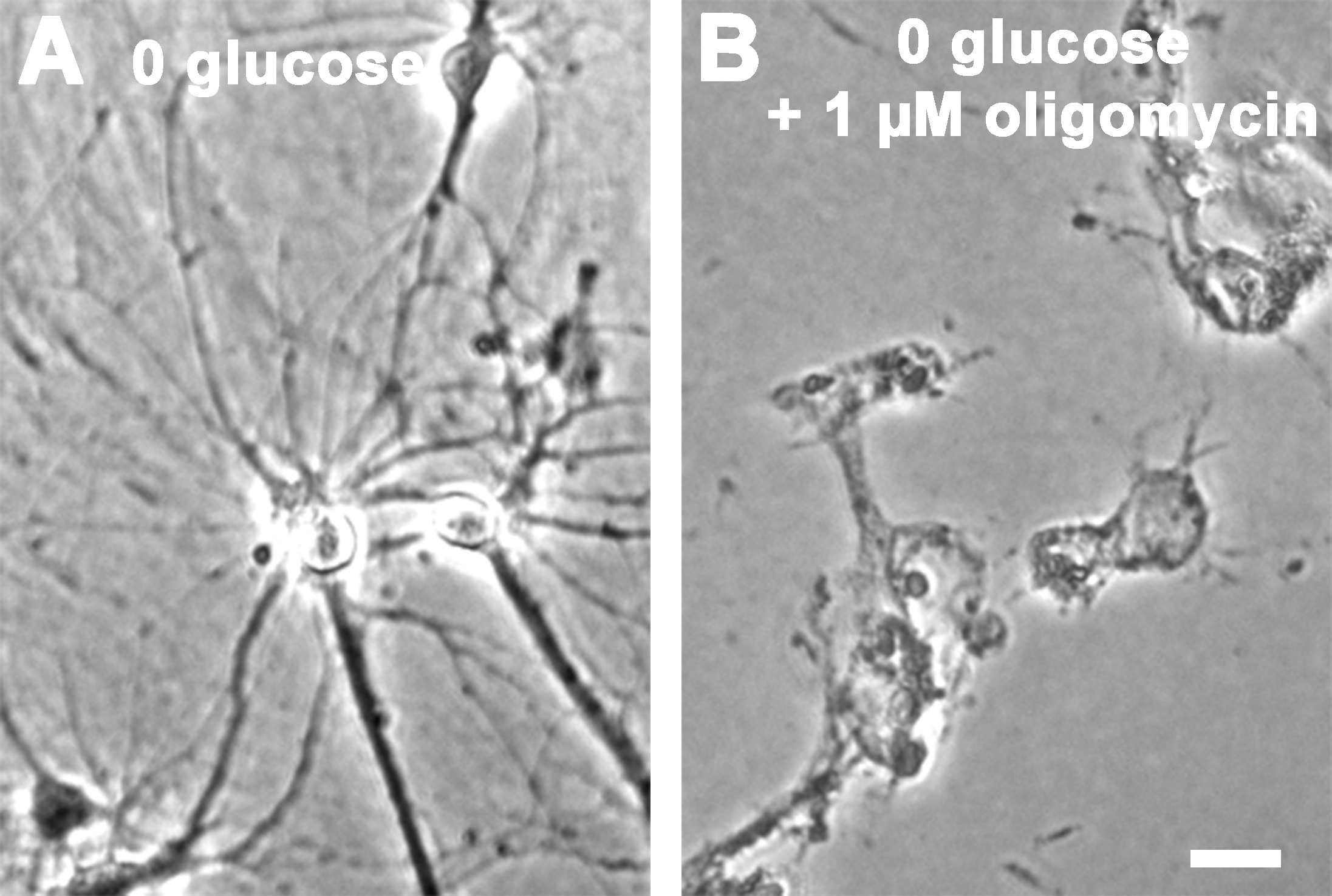

Supplement: S3 Fig — A. Survival of neurons following glucose deprivation. B. Neurons and most astrocytes were eliminated by addition of 1 μM oligomycin to the incubation solution. Photomicrographs are representative of 3 independent experiments. Scale bar, 25 μm. (TIF) [file pone.0195520.s003.tif]

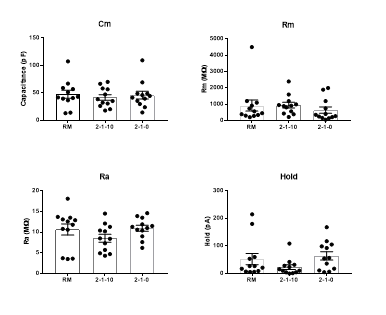

Supplement: S4 Fig — Experimental conditions were 0 glucose and 10 mM glucose conditions were incubated in defined saline solution containing the indicated glucose for 16 h prior to recording in the same glucose condition. No effect of glucose deprivation was detected on any parameter. Holding current (Hold) was current at -70 mV. (TIF) [file pone.0195520.s004.tif]

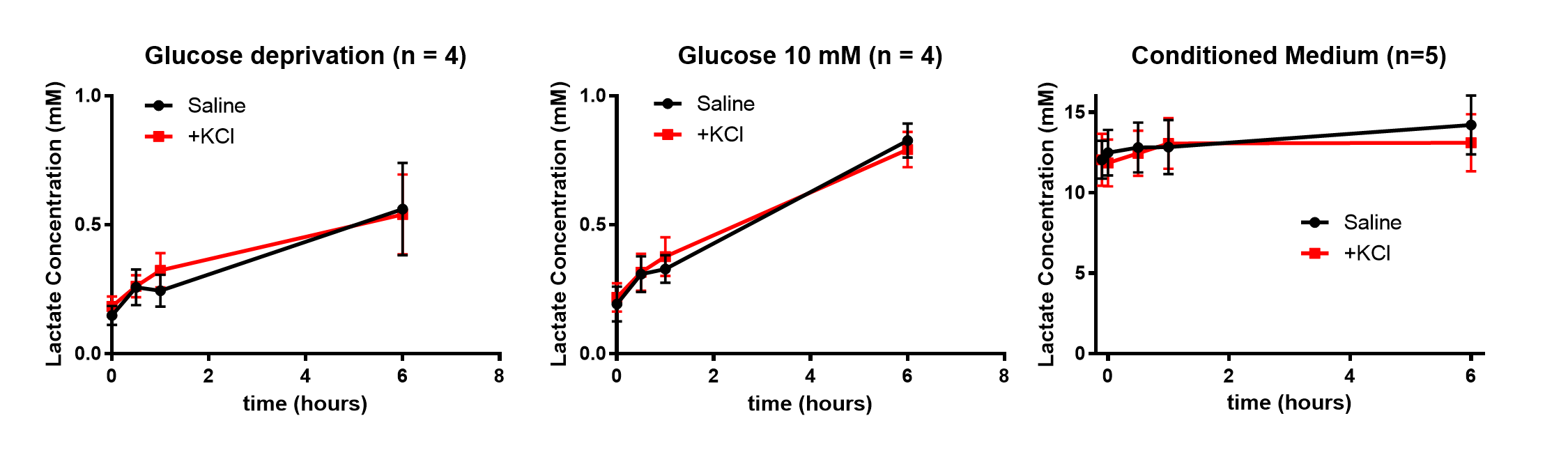

Supplement: S5 Fig — (TIF) [file pone.0195520.s005.tif]

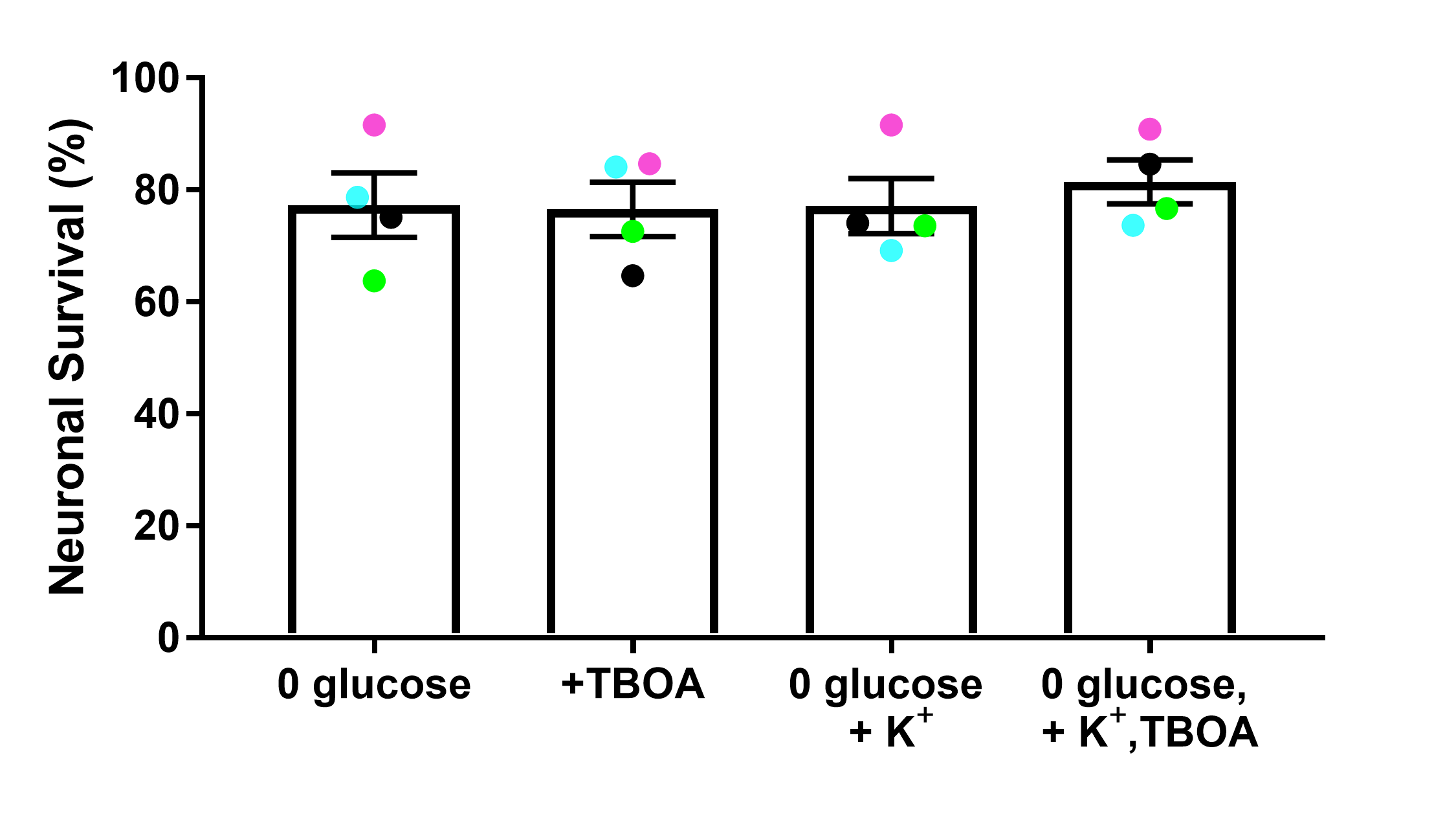

Supplement: S6 Fig — Incubations in all treatments were for 16 h. K+ concentration was 30 mM. (TIF) [file pone.0195520.s006.tif]
